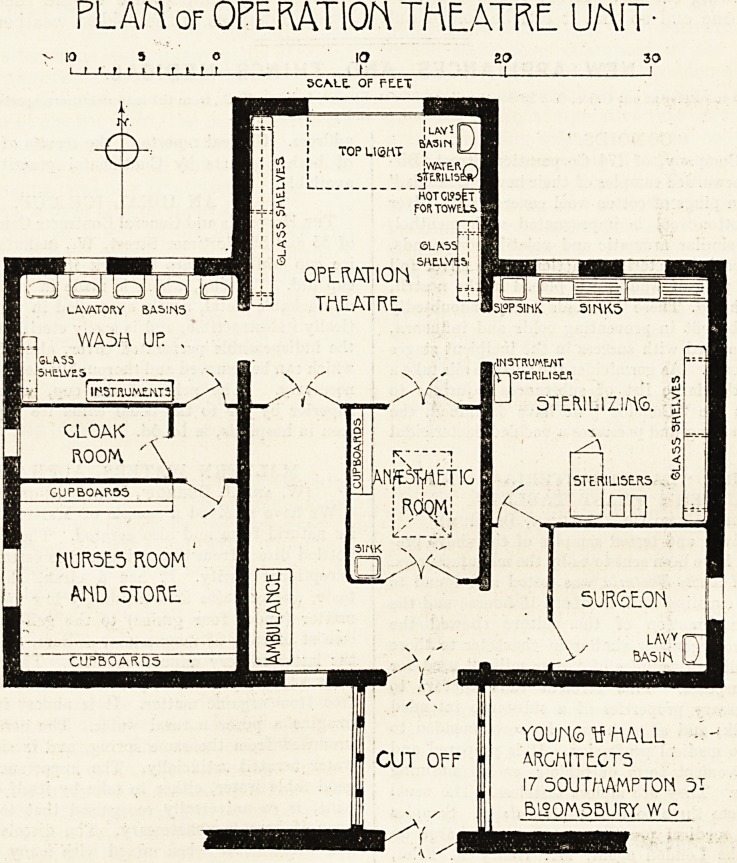# The Operation Theatre

**Published:** 1909-10-16

**Authors:** 


### THE OPERATION THEATRE.

### III.—THE THEATRE UNIT.

The theatre unit for a small hospital where only one operation theatre is required must of necessity be proportionately larger than in the case of a hospital where two or more theatres are needed; for whereas a single theatre must be provided with its full complement of accessory rooms, where there are several theatres grouped together as at St. Thomas's or the London Hospital, some, at any rate, of these rooms may serve for the whole set of theatres. If, however, a hospital is planned on the unit system, with a separate theatre to each unit, then to each theatre the full complement of rooms will be required. Our plan shows a theatre unit suitable for the smallest class of general hospital, and where no provision for spectators is required. The whole block is cut off from the main hospital by a corridor provided with cross ventilation; double doors of ample width to admit the passage of an ambulance are placed at each end of a lobby, which thus serves the double purpose of an airlock and of preventing the passage of sound from the theatre to the main corridor. The doors at the theatre end of the lobby open into a vestibule, around which the various rooms are placed.

### The Anæsthetic Room.

Immediately opposite the lobby doors is the entrance to the anæsthetic room. The position of this room is carefully devised so that a patient is wheeled directly into it and from it into the theatre, while, after the operation, he leaves the theatre by a different door and so does not pass through the anæsthetic room again. This is a detail of importance as it may often happen that there is a second patient waiting in the anæsthetic room when the first is wheeled out of the theatre. The doors into the theatre and out of the theatre into the corridor are wide enough to allow of the passage of the ambulance, with, if necessary, an attendant walking on each side. In the anæsthetic room there should be a cupboard for holding the necessary apparatus used for administering anæsthetics, a small sink provided with hot and cold water, and a desk on which to place the record book of anæsthetics.

The Theatre.

The theatre occupies a central position in the block and faces due north. It is lighted in the way described in the preceding article, that is by a large vertical window 8 feet wide, continuous with which is a roof-light the same width and 6 feet long. This arrangement practically gives an ample uninterrupted area of light. The fittings in the theatre comprise a lavatory basin for the surgeons, a steam apparatus for sterilised water, a small steam-heated closet for towels, and a provision of glass shelves to stand porringers and trays and the various other things that are needed during an operation.

The Sterilising Room.

On the east side of the theatre is the sterilising-room, and on the west side is the washing-room for house surgeons and nurses. In the sterilising-room we have placed the slop sink. It seems very desirable that this fitting should be outside the theatre, but at the same time it is most essential that it should be within easy reach of the theatre. It is, therefore, close to the doorway between the theatre and the sterilising-room. On the other side of the doorway and closely connected with the theatre is the instrument steriliser. It is very necessary that this fitting should be within ready access of the theatre as it does happen occasionally that an instrument has to be sterilised during the course of an operation. The other fittings in this room comprise a steam-jacketed steriliser of the Lyons type for dressings, coats, and other things for which dry heat is essential, two steam sterilisers for bowls and trays, two porcelain sinks for washing bowls and other vessels, each with a large glass draining-board and a suitable provision of glass shelves on which to put sterilised articles.

Other Rooms in Unit.

The wash-room for the house surgeons and nurses contains five wash-hand basins, glass shelving for the operating coats, veils, etc., and the instrument-cabinet. To the right of the entrance is a small room for the surgeon provided with a lavatory-basin. On the opposite side is a room for the nurse in charge of the department which is fitted with cupboards for linen, crockery, and other things which it is necessary to keep within close neighbourhood of the theatre. Adjoining this is a small cloak-room where the house surgeons leave their coats.

### Heating and Ventilation.

Ample provision must, of course, be made for warming and ventilating the whole of the rooms of this department. The simplest method of warming is that of hot-water radiators with admission of fresh air in connection with each. For extract ventilation there should be a suitably proportioned trunk from each room, all of which may be taken into one main shaft in the roof and provided with a fan for expelling the air. Every radiator should be pivoted at one end so that it can be folded out and thoroughly cleansed in every part, and also that the wall and the fresh-air inlet can be cleaned. The inlets should each be provided with a suitable filtering material. For the ventilation of the theatre itself it is better not to rely solely on extraction, but to provide in the outer wall immediately under the window a fan to force the air in. The incoming air will pass over the pipes of the radiator and be warmed in its passage into the room. Care, of course, must be taken to provide ample heating surface in this radiator so that the proper temperature can always be kept up. It should always be possible to keep the temperature of the theatre up to at least 70 degrees in the coldest weather.

## Figures and Tables

**Figure f1:**